# Supplementary material for: Growing urban bicycle networks
Source: Sci Rep. 2022 Apr 26;12:6765. doi: 10.1038/s41598-022-10783-y (PMC9039277; doi:10.1038/s41598-022-10783-y)
Supplement: Supplementary file 1 — Supplementary Information. [file 41598_2022_10783_MOESM1_ESM.pdf]

Growing Urban Bicycle Networks
Supplementary Information

Michael Szell[1,2,3], Sayat Mimar[4], Tyler Perlman[4], Gourab Ghoshal[4], and Roberta Sinatra[1,2,3]

[1] *NEtwoRks, Data, and Society (NERDS), IT University of Copenhagen, 2300 Copenhagen, Denmark*
[2] *ISI Foundation, 10126 Turin, Italy*
[3] *Complexity Science Hub Vienna, 1080 Vienna, Austria*
[4] *Department of Physics and Astronomy, University of Rochester, Rochester, NY 14627, USA*

This is the supplementary information for the manuscript containing supplementary notes, figures, and tables.

List of Figures

1 Square and triangle for network coverage calculations. . . . 3
2 Synthetic versus real bicycle networks. . . . 6
3 Directness definitions 6
4 Streets: Change of network metrics with growing bicycle networks. . . . 7
5 Change of network metrics with betweenness growth on rail station seeds. . . . 8
6 Change of network metrics with closeness growth on rail station seeds. . . . 9
7 Change of network metrics with random growth on rail station seeds. . . . 10
8 Change of network metrics with betweenness growth on grid seeds. . . . 11
9 Change of network metrics with closeness growth on grid seeds. . . . 12
10 Change of network metrics with random growth on grid seeds. . . . 13

List of Tables

1 Cities and their urban parameters. . . . 5

Supplementary Note 1: Limitations and refinements for planning

Since we publish all our code as open source, arbitrary refinements are possible, expected, and encouraged in future work, especially towards better applicability of our approach for concrete bicycle network planning. For example, the CROW manual recommends a hierarchical network approach with a top-level bicycle highway connecting the major neighborhoods, a medium-level "main cycle network", and a low-level "basic infrastructure" [14]. In our process we only considered the medium level so far, but it is straightforward to place custom links before or after the greedy triangulation which allows to create any desired hierarchy. To operationalize CROW's "adapted grid method" [14] even closer, a quadrangulation [81] could be implemented for grid-like seed points and streets, especially for US cities [79], as well as the missing harmonization with other transport layers. However, computational difficulties must be expected with quadrangulations since in general they are not guaranteed to have a solution and are computationally less feasible [81].

Further, our approach does not consider bicycle network planning outside of built-up urban areas, which is becoming increasingly pressing with the rise of affordable e-bike technologies. This line of research is also becoming more important due to large-scale bicycle planning initiatives such as Denmark's infrastructure plan for 2035, where 2-3 billion DKK are to be invested into national bicycle infrastructure, primarily into cycle superhighways. The development of inter-urban, regional networks demands different considerations outside the scope of our paper, from changed parameters or topology such as "ladder structures" [14] to accounting for different regional stakeholders and investors.

The biggest limitation of our approach is the sole focus on retrofitting street networks for safe cycling. This approach has some issues because it only considers on-street but no off-street bicycle infrastructure – in particular, our algorithms ignore existing bicycle-only infrastructure such as Copenhagen's Cykelslangen or similar bicycle-only infrastructure in many parts of the Netherlands – but this issue is only relevant in such well developed cycling environments and negligible in the vast majority of cities on the planet. The first main problem is about low-density regions. Such regions, like most parts of the Netherlands, rightly prioritize the development of off-street networks to keep cycle routes as far as possible from cars, avoiding intersecting traffic, thus making them attractive low-stress environments [14]. Therefore, although our approach is the only feasible for high-density cities, in low-density urban environments a Dutch style off-street approach can be argued to be more desirable than on-street networks. For example, although Copenhagen has a very well developed on-street bicycle network with a high modal share for cycling, it is questionable 1) to which extent such a cycling environment is low-stress, inclusive, and child-friendly, and 2) whether such on-street tracks are literally cementing the "arrogant" car-centric distribution of mobility space that has indeed been reported in Copenhagen [6]. Thus, a focus on building on-street networks possibly represents a lock-in into a path-dependent city evolution that could complicate the transition to car-free cities, therefore being unsustainable in the long term [2]. Second, an exclusion of off-street infrastructure also could exclude improved geometric solutions such as Steiner trees/points [81]. Third, from a communication perspective, this approach comes with the risk of anchoring automobile infrastructure as the default when in reality automobile transport is one of the most unsustainable and possessive modes (in terms of public space demands) for cities [5] posing the biggest threat to the lives of other road users [19]. For all these reasons, future research on bicycle network growth should consider and prioritize off-street solutions where possible.

Our minimal requirements on data, making use of only the street network, could be seen as another limitation. However, we follow this approach on purpose for our framework to be applicable to data-scarce environments, and thus to a large part of the planet [41]: no lane widths, inclines, traffic flows, etc. are needed to optimize geometry. Further, even if we had for example cyclist flows available, how would we ensure they are a ground truth for optimal infrastructure? Such flow data could have been influenced by inappropriately or suboptimally developed cycling infrastructure and other biases. Thus, our research approach places itself on the right end of the spectrum: Single city with complex/multiple data sets — Multiple cities with simple/few data sets.

Given that the struggle to implement more bicycle infrastructure is mostly a political one, it could be argued that there might already be enough theoretical knowledge generated by cycling research. While there is a political bottleneck, the questions of *How* to do provide more road space for cyclists at the cost of motorized traffic and of *Why* that is happening so slowly cannot be neglected. Our study shows that cycling research has barely scratched the surface when it comes to understanding the underlying network effects, given that network percolation and its fundamental implication on network growth is one of the most basic findings of graph theory dating back to the 1950s [36] but is not common knowledge in transport network planning – otherwise it would be acknowledged in leading planning manuals such as CROW [14]. Last but not least, research and politics are not independent: If cycling research is becoming "mainstream" and is accompanied by high-quality visualizations, open source tools, and a compelling vision, it can create increased potential to reach the general public and induce political change [5, 22, 68].

Supplementary Note 2: Grid size and network coverage

The grid points in the grid seed network triangulation were chosen at a distance of $a_\triangle = 1707\,\text{m}$ ensuring full coverage of the city when $r_{\max} = 500\,\text{m}$ is assumed as a reasonable maximum distance (around 5 minutes of walking). The proof for this coverage comes from the fact that the right triangle with sides of lengths $a_\triangle$, $a_\triangle$ and $\sqrt{2}a_\triangle$ is the triangle that emerges in the triangulation, together with its inradius equation $r_{\max} = \frac{a_\triangle + a_\triangle - \sqrt{2}a_\triangle}{2}$. Solving for $a_\triangle$ yields $a_\triangle = \frac{2}{2-\sqrt{2}} r_{\max} \approx 3.41 r_{\max}$. Comparing this argumentation to a square grid, which is the network geometry suggested by the CROW manual, a maximum distance of $r_{\max} = 500\,\text{m}$ would correspond to a grid length of $a_\square = 2r_{\max} = 1000\,\text{m}$. The grid size standard given by CROW for built-up areas is 300-500 m. If a similarly tightly covering mesh is desired, our grid triangulation would need to be run for a grid with half to third of its grid length.

Instead of the maximum distance $r_{\max}$, it is more useful for planning purposes to consider the average distance r_{avg} of a random point to the network. As we show in the calculations below, the average distance for our choice $a_\triangle = 1707\,\text{m}$ corresponds to $r_{\text{avg}} = r_{\max}/3 \approx 167\,\text{m}$. For a square CROW grid of length 500 m the average distance would be $r_{\text{avg}} \approx 83\,\text{m}$. These numbers are only crow flies distances and do not account for less direct distances routed on concrete street or pedestrian networks – to account for street grid effects all distance values could be multiplied by up to $\sqrt{2}$. In other words, the effective average distance to the network could be closer to $\sqrt{2} \cdot 167 \approx 236\,\text{m}$ than to 167 m.

Calculation of average distance to the nearest side of a square

What is of interest to us is to calculate the average distance from a randomly selected interior point to the side of the square closest to it. By subdividing the square into triangles by drawing the line segments connecting each corner to the center, all points for which a given sides is the closest will be in the same sub triangle. From there, we can integrate the distances to the closest side over each of these triangles, sum these values, and divide by the total area of the square to get our desired result.

For a square with side length $a_\square$, if we were to align two of the with the axes, our center would be at $(\frac{a_\square}{2}, \frac{a_\square}{2})$. The lines that connect the corners to the center (the diagonals) would be given as follows, see Supplementary Figure 1 (left):

$$f(x) = x$$
$$g(x) = -x + a_\square.$$

First, by noting that all of our sub-triangles are congruent, isosceles triangles, we can further divide our triangles along the altitudes from each side of the square to the center. This results in a new count of 8 congruent, isosceles triangles for which all interior points of each triangle are closest to the same side of the square. For the triangles with sides on the x-axis, the distance from an interior point to the closest edge is the point's distance to the x-axis. As all of our triangles are congruent, we need to calculate one integral:

$$I = \int_0^{a_\square/2} \left(\int_0^{f(x)} y dy \right) dx$$

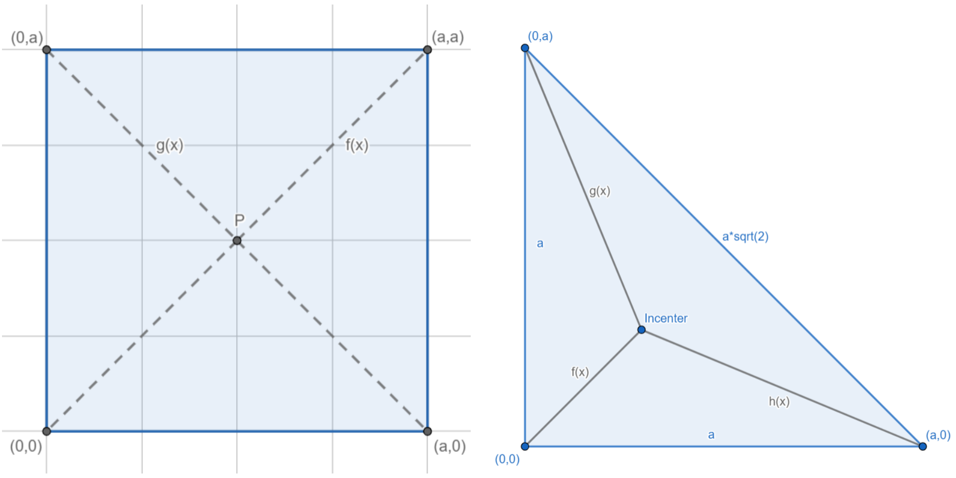

Supplementary Figure 1: **Square and triangle for network coverage calculations.**

Evaluating this integrals yields:

$$I = \frac{a_\square^3}{48}.$$

Thus, the average distance from an interior point of the square to the nearest edge will be: $I \div \frac{a_\square^2}{8} = \frac{a_\square^3}{48}\frac{8}{a_\square^2} = \frac{a_\square}{6}$. As such:

$$r_{\text{avg}} = \frac{1}{6}a_\square$$

Calculation of average distance to the nearest side of an isosceles triangle

What is of interest to us is to calculate the average distance from a randomly selected interior point to the side of the triangle closest to it. By finding the incenter of the circle (the point equidistant from all sides) and subdividing the triangle by drawing the line segments connecting each corner to the incenter, all points for which a given sides is the closest will be in the same triangle. From there, we can integrate the distances to the closest side over each of these triangles, sum these values, and divide by the total area of the triangle to get our desired result.

For a right isosceles triangle with side lengths $a_\triangle, a_\triangle,$ and $a_\triangle\sqrt{2}$, the inradius is $r = \frac{2-\sqrt{2}}{2}a_\triangle$. As such, if we were to align the sides that intersect at a right angle with the axes, our incenter would be at (r, r). The lines that connect the corners to the incenter (and thus bisect each of the angles) would be given as follows, see Supplementary Figure 1 (right):

$$\begin{aligned} f(x) &= x \\ g(x) &= -(1+\sqrt{2})x + a_\triangle \\ h(x) &= (1-\sqrt{2})x - (1-\sqrt{2})a_\triangle. \end{aligned}$$

First, we can simplify the problem: by noting that as the triangle is isosceles, we can cut the triangle in half along the line $y = x$ and calculate our average distance over one of these halves of the triangle. We then take the part of the sub-triangle for which the interior points are closest to hypotenuse, and rotating our coordinates so that the hypotenuse is on the x-axis. By doing so, for both this triangle as well as the other sub-triangle, the distance from an interior point to the closest edge is the point's distance to the x-axis. We will also subdivide the second triangle, but breaking it up along the altitude from the incenter to the edge. As such, our integrals of interest are:

$$\begin{aligned} I_1 &= \int_0^r \left(\int_0^{f(x)} y dy\right) dx \\ I_2 &= \int_r^{a_\triangle} \left(\int_0^{h(x)} y dy\right) dx \\ I_3 &= \int_0^{a_\triangle \frac{\sqrt{2}}{2}} \left(\int_0^{(\sqrt{2}-1)x} y dy\right) dx \end{aligned}$$

where $y = (\sqrt{2}-1)x$ is the line connecting $(0, 0)$ to $(a\frac{\sqrt{2}}{2}, r)$. It is worth noting that the sub-triangles corresponding to I_2 and I_3 are congruent, and as such, these integrals will be equal. Evaluating these integrals yields:

$$\begin{aligned} I_1 &= \frac{1}{48}\left(2-\sqrt{2}\right)^3 a_\triangle^3 \\ I_2 &= \frac{1}{24}\left(3\sqrt{2}-4\right) a_\triangle^3 \\ I_3 &= \frac{1}{24}\left(3\sqrt{2}-4\right) a_\triangle^3. \end{aligned}$$

Thus, the sum of these integrals will be: $I_1 + I_2 + I_3 = \frac{1}{24}\left(2-\sqrt{2}\right) a_\triangle^3$. Now, we divide by half of the area of the triangle, $\frac{a_\triangle^2}{4}$. This yields:

$$r_{\text{avg}} = \frac{2-\sqrt{2}}{6}a_\triangle$$

City	L_{car} [km]	L_{bike} [km]	Γ_{bike}	Population	Area [km^2]	Continent	Density [Pop/km^2]
Tokyo	26,293	339	504	37,977,000	8,547	Asia	4,443
Jakarta	11,797	4	15	34,540,000	3,225	Asia	10,710
Mumbai	3,983	2	1	23,355,000	546	Asia	42,775
Sao Paulo	17,029	250	127	22,046,000	2,707	S. America	8,144
Mexico City	15,056	247	63	20,996,000	2,072	N. America	10,133
Moscow	5,507	286	229	17,125,000	4,662	Europe	3,673
Buenos Aires	3,399	257	57	16,157,000	2,681	S. America	6,026
Karachi	20,272	0	1	14,835,000	945	Asia	15,698
Los Angeles	12,286	292	276	12,750,807	6,299	N. America	2,024
Paris	1,536	292	314	11,020,000	2,845	Europe	3,873
London	15,810	1,236	4,196	10,979,000	1,738	Europe	6,317
Bogota	6,788	471	179	9,464,000	492	S. America	19,236
Chicago	7,003	163	160	8,604,203	6,856	N. America	1,255
Luanda	1,878	0	0	8,417,000	894	Africa	9,415
Hong Kong	3,692	272	329	7,347,000	275	Asia	26,716
Singapore	4,502	470	249	5,745,000	518	Asia	11,091
Philadelphia	4,576	109	70	5,649,300	5,131	N. America	1,101
Houston	13,120	287	97	5,464,251	4,644	N. America	1,177
Toronto	6,275	299	183	5,429,524	2,287	N. America	2,374
Boston	1,744	124	86	4,688,346	5,325	N. America	880
Barcelona	1,412	217	79	4,588,000	1,075	Europe	4,268
Phoenix	9,537	341	107	4,219,697	3,196	N. America	1,320
Berlin	6,181	1,334	803	3,644,826	1,347	Europe	2,706
San Francisco	1,847	76	115	3,592,294	2,797	N. America	1,284
Montreal	5,687	362	165	3,519,595	1,546	N. America	2,277
Detroit	5,108	75	44	3,506,126	3,463	N. America	1,012
Birmingham	2,774	237	265	2,897,303	598	Europe	4,845
Rome	7,282	214	190	2,872,800	1,114	Europe	2,579
Greater Manchester	9,836	668	797	2,705,000	1,277	Europe	2,118
Tashkent	3,633	4	9	2,424,100	334	Asia	7,258
Leeds	3,290	247	425	1,901,934	487	Europe	3,905
Hamburg	4,349	858	885	1,841,179	755	Europe	2,439
Vienna	3,023	573	478	1,840,573	414	Europe	4,446
Warsaw	3,649	630	217	1,790,658	517	Europe	3,464
Budapest	4,507	210	206	1,752,286	525	Europe	3,338
Manhattan	972	152	77	1,628,706	87	N. America	18,721
Rabat	1,152	4	7	1,628,000	117	Africa	13,915
Munich	2,785	1,080	466	1,471,508	310	Europe	4,747
Ulaanbaatar	6,180	24	14	1,396,288	4,704	Asia	297
Milan	1,964	185	230	1,351,562	181	Europe	7,467
Cologne	2,720	764	427	1,085,664	405	Europe	2,681
Copenhagen	1,159	454	221	1,085,000	292	Europe	3,716
Amsterdam	1,770	718	288	1,031,000	219	Europe	4,708
Glasgow	2,088	147	189	985,290	142	Europe	6,939
Kathmandu	808	3	21	975,453	49	Asia	19,907
Marrakesh	1,860	2	3	928,850	143	Africa	6,495
Turin	1,643	171	100	870,952	130	Europe	6,700
Oslo	1,737	294	555	693,494	480	Europe	1,445
Sheffield	2,017	103	233	685,368	368	Europe	1,862
Helsinki	1,572	1,299	506	642,045	715	Europe	898
Stuttgart	1,526	161	334	634,830	207	Europe	3,067
Shah Alam	3,079	57	15	584,340	290	Asia	2,015
Bradford	1,985	59	102	540,000	370	Europe	1,459
Lyon	653	62	116	516,092	48	Europe	10,752
Edinburgh	1,623	162	199	488,050	264	Europe	1,849
Tel Aviv	813	163	195	451,523	52	Asia	8,683
Zurich	764	64	330	434,008	87	Europe	4,989
Malmo	919	428	142	321,845	332	Europe	969
Santiago Centro	399	48	9	200,792	22	S. America	9,126
Bern	408	11	89	133,798	51	Europe	2,623
Delft	285	111	145	103,000	24	Europe	4,292
Bath	272	17	32	88,859	29	Europe	3,064

Supplementary Table 1: **Cities and their urban parameters.**

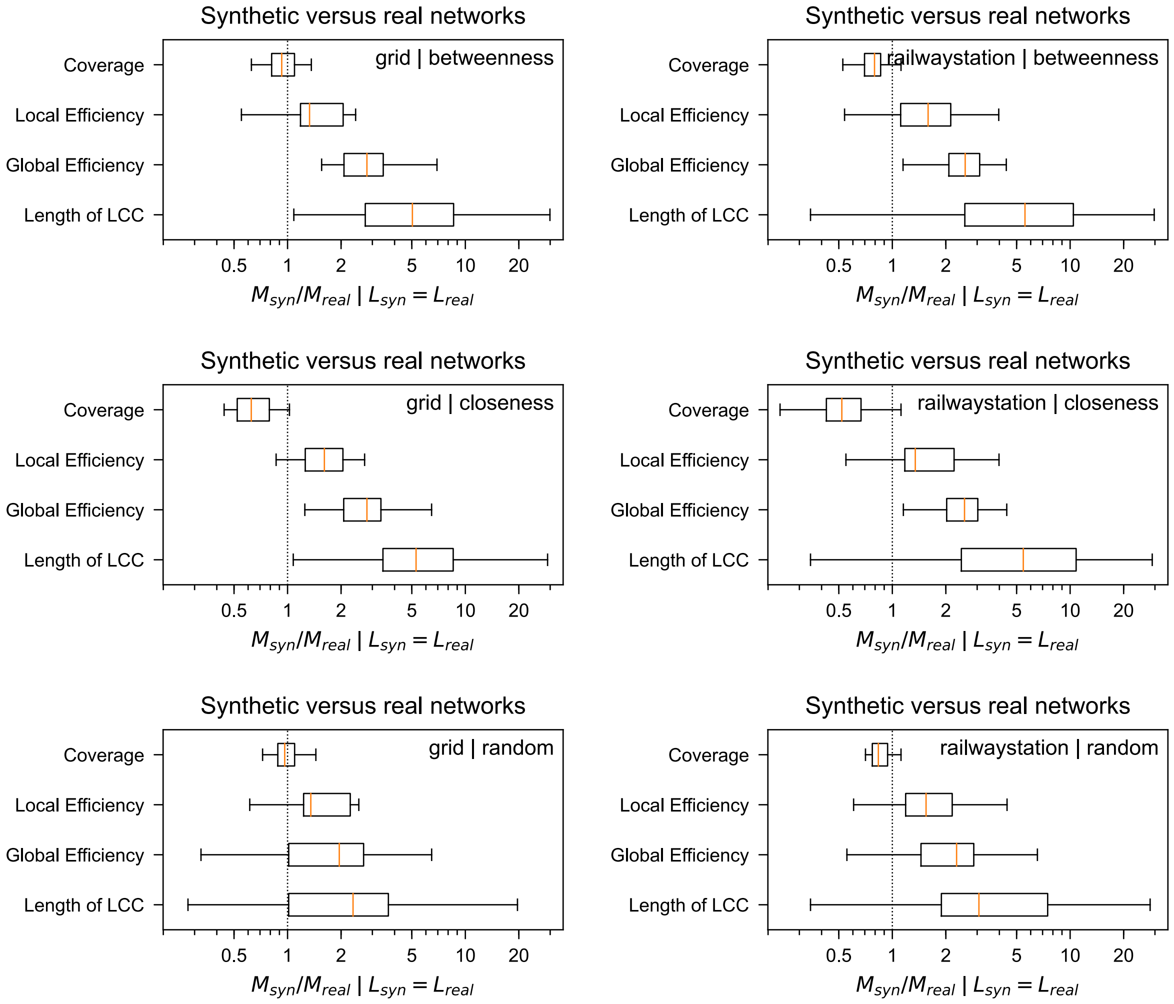

Supplementary Figure 2: **Synthetic versus real bicycle networks.** Shown are all 6 combinations of growth strategies (betweenness, closeness, random) with seed types (grid, rail).

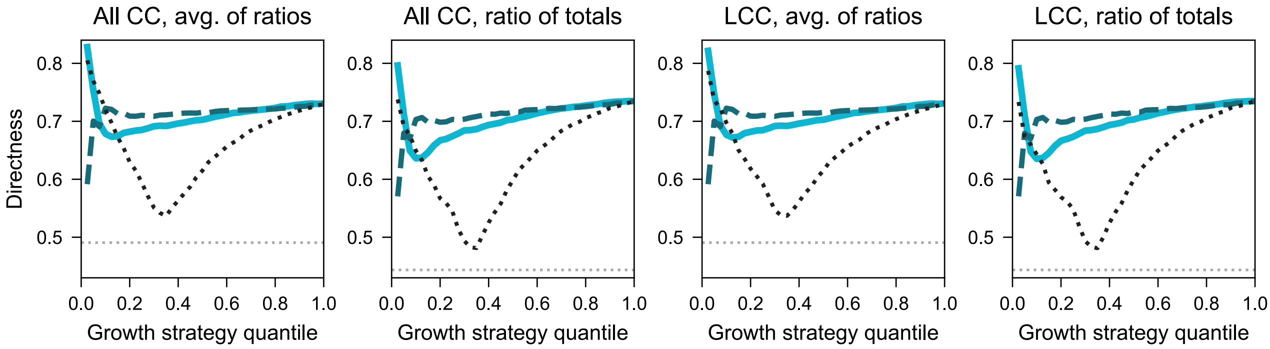

Supplementary Figure 3: **Comparison of directness definitions.** The four different definitions of directness, see Methods in the main text, averaged over all 62 cities for grid seeds. The plots show that numerical values vary only insignificantly; the results are qualitatively identical for each definition. For the legend see Fig. 4 in the main text.

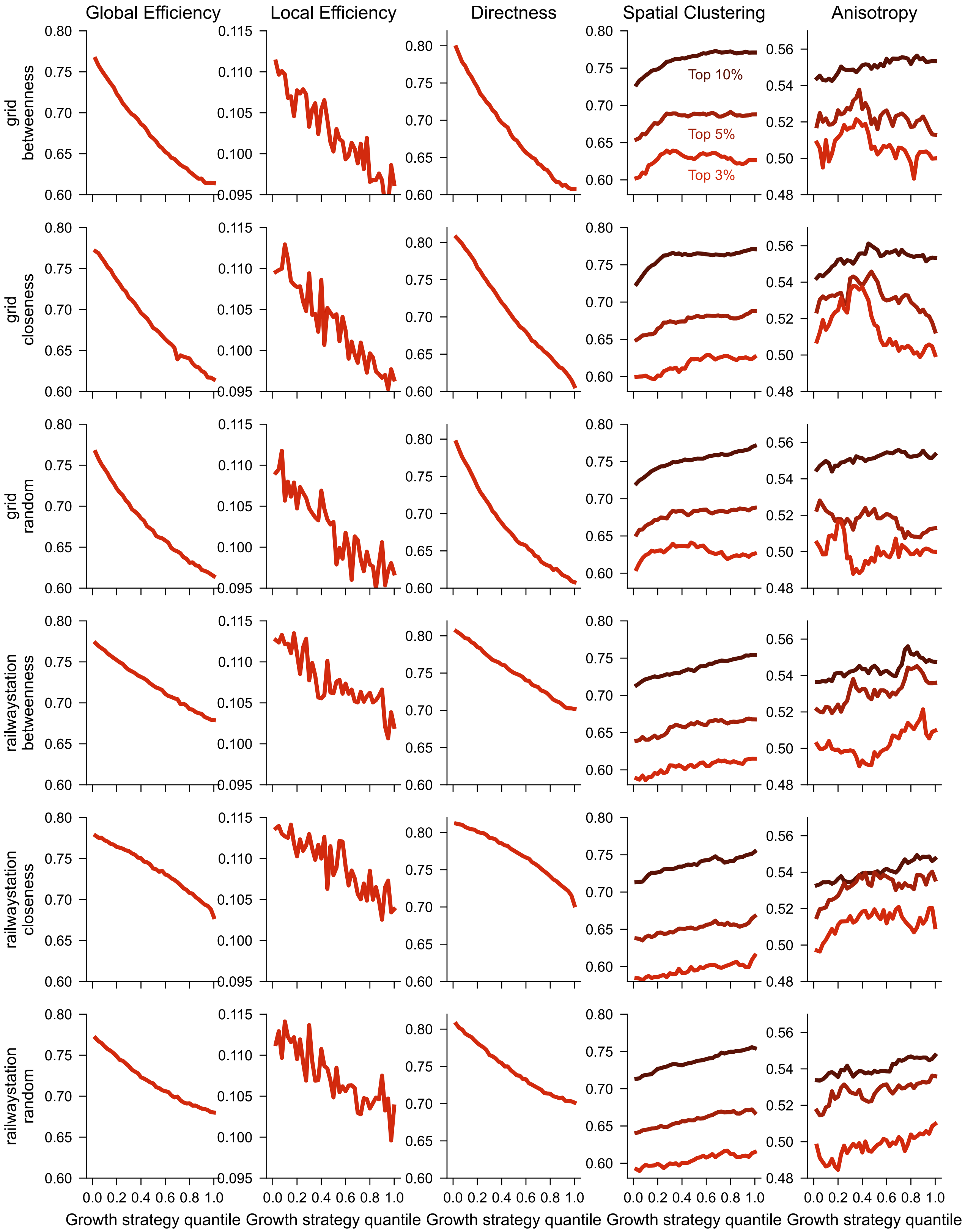

Supplementary Figure 4: **Streets: Change of network metrics with growing bicycle networks.**

Copenhagen (railwaystation | betweenness)

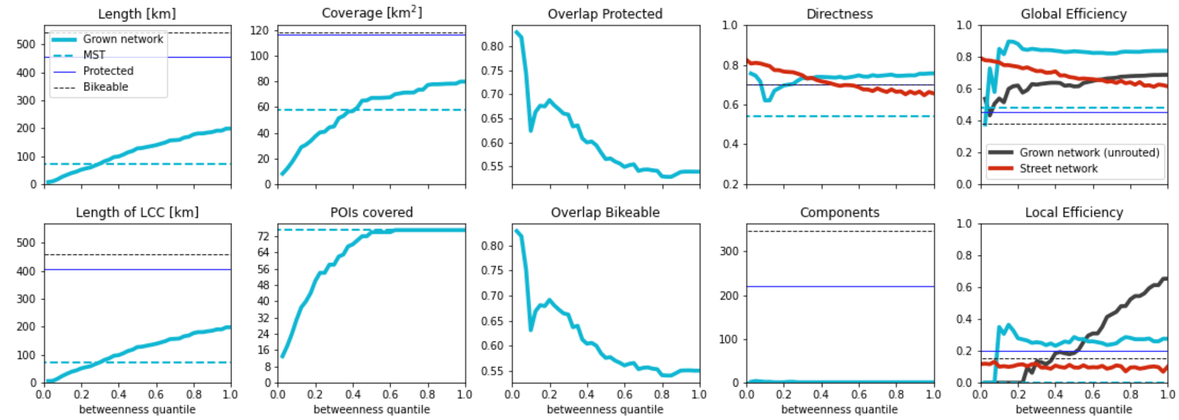

Boston (railwaystation | betweenness)

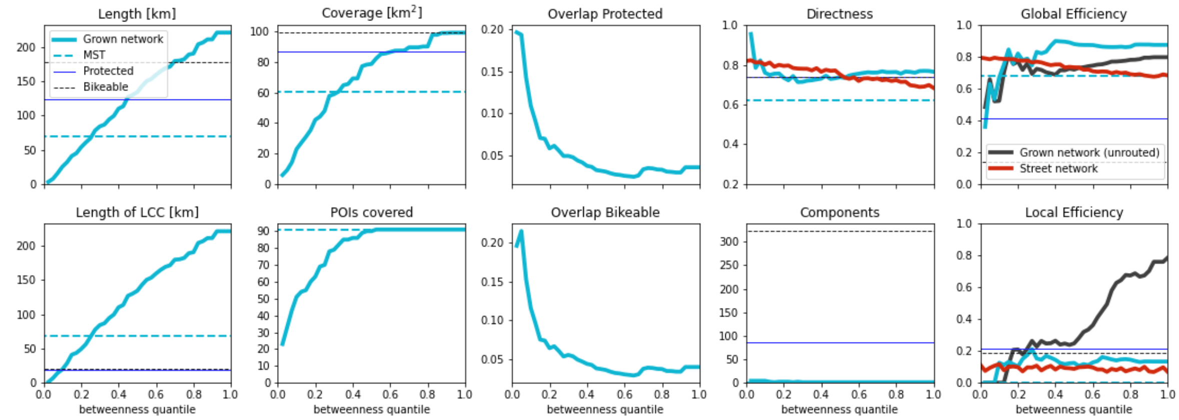

Mumbai (railwaystation | betweenness)

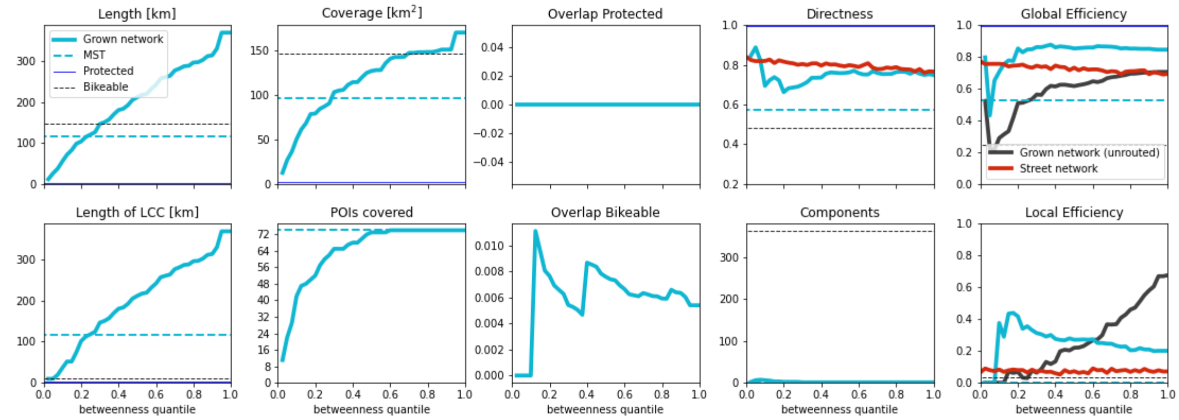

Tokyo (railwaystation | betweenness)

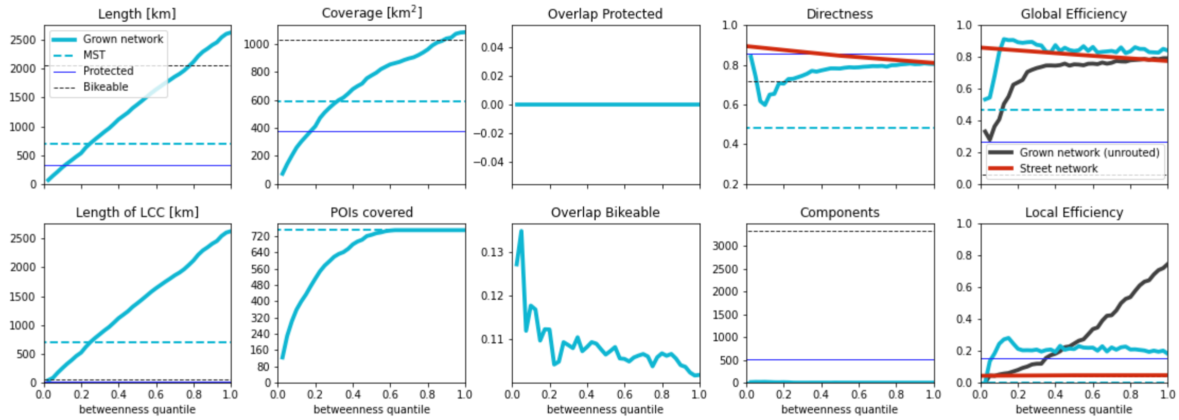

Supplementary Figure 5: **Change of network metrics with betweenness growth on rail station seeds.** Shown are the selected cities Copenhagen, Boston, Mumbai, Tokyo. See Section Data availability in the main text for how to access these plots for all cities.

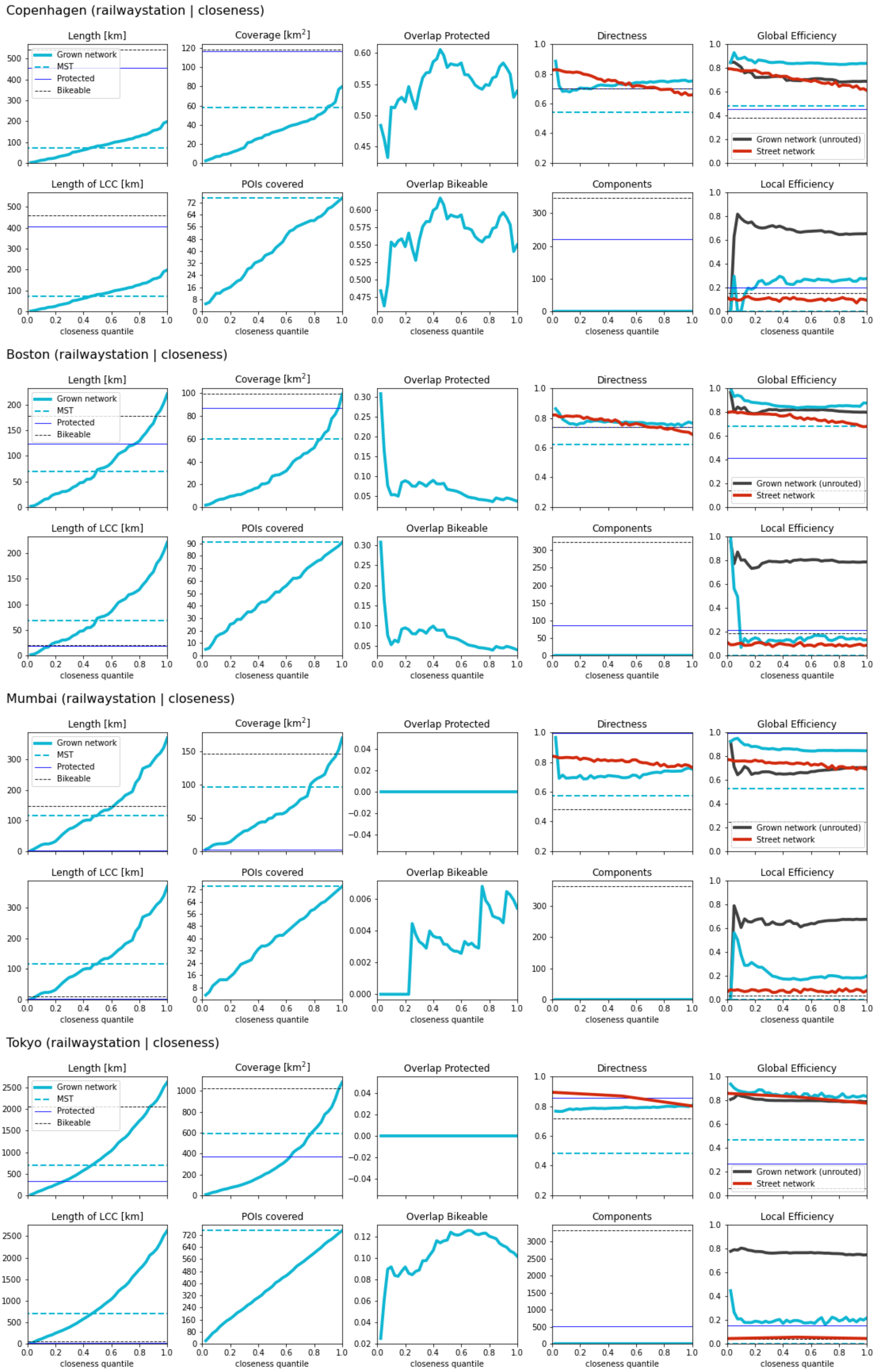

Supplementary Figure 6: **Change of network metrics with closeness growth on rail station seeds.** Shown are the selected cities Copenhagen, Boston, Mumbai, Tokyo. See Section Data availability in the main text for how to access these plots for all cities.

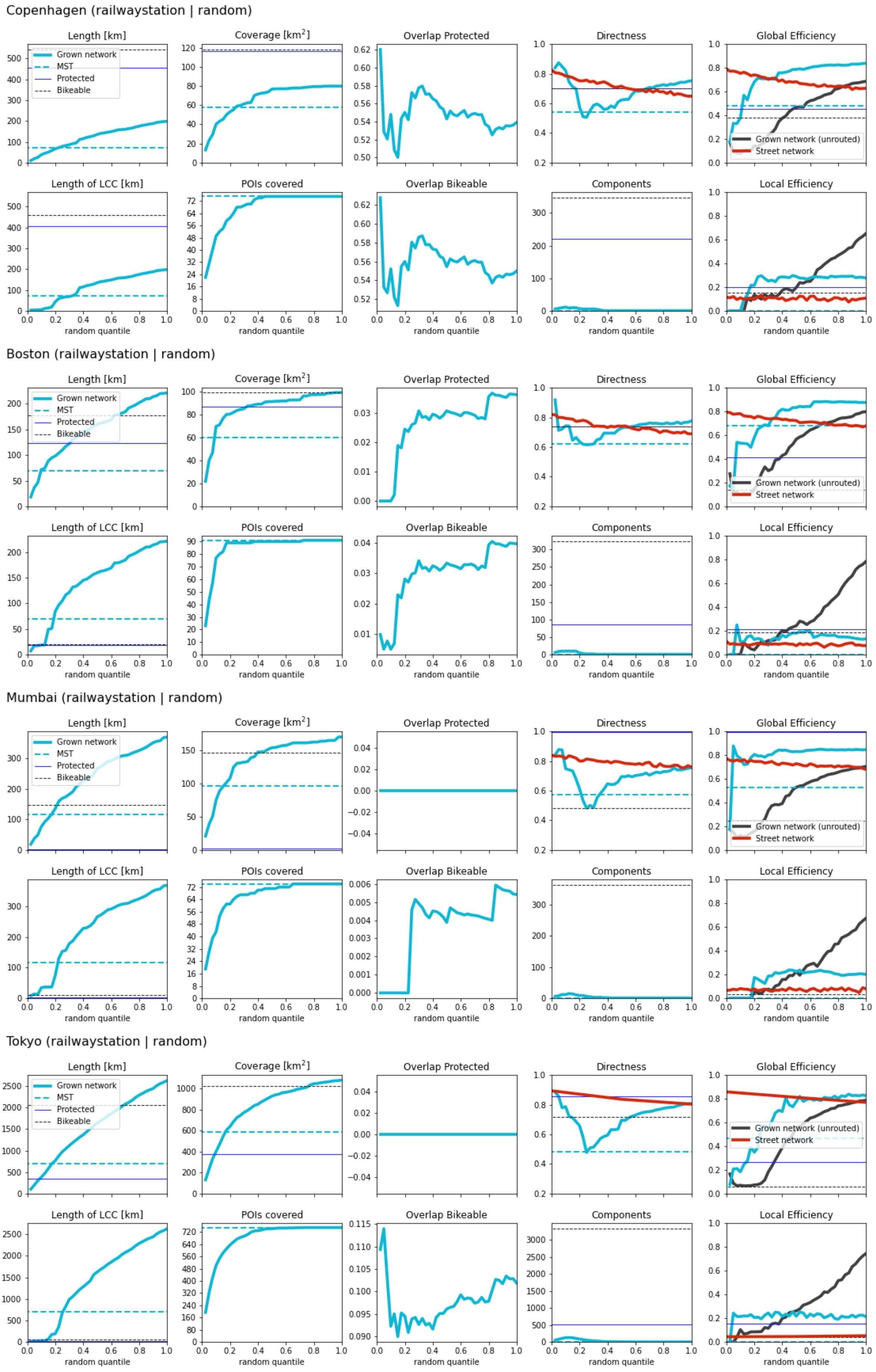

Supplementary Figure 7: **Change of network metrics with random growth on rail station seeds.** Shown are the selected cities Copenhagen, Boston, Mumbai, Tokyo. See Section Data availability in the main text for how to access these plots for all cities.

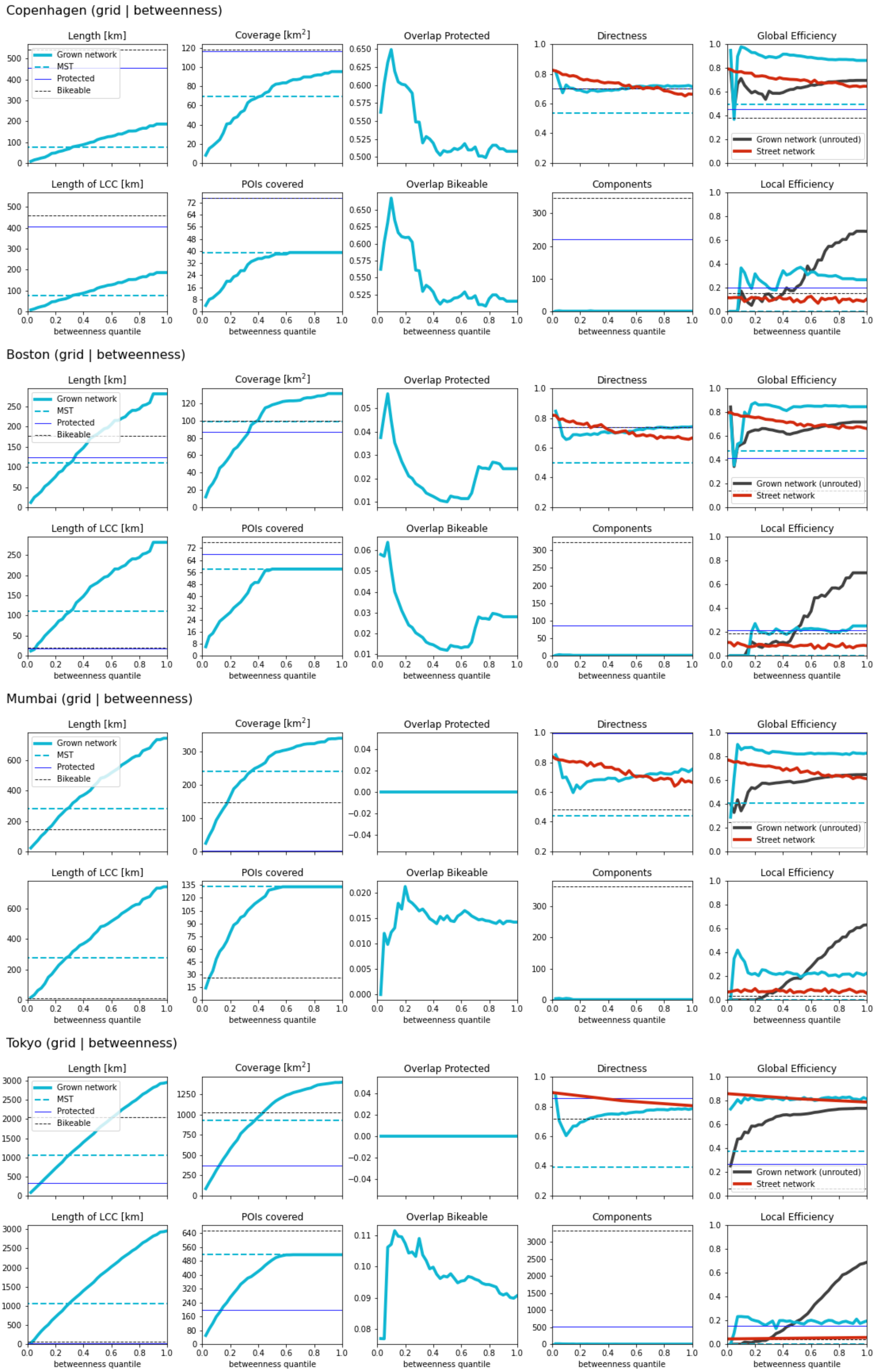

Supplementary Figure 8: **Change of network metrics with betweenness growth on grid seeds.** Shown are the selected cities Copenhagen, Boston, Mumbai, Tokyo. See Section Data availability in the main text for how to access these plots for all cities.

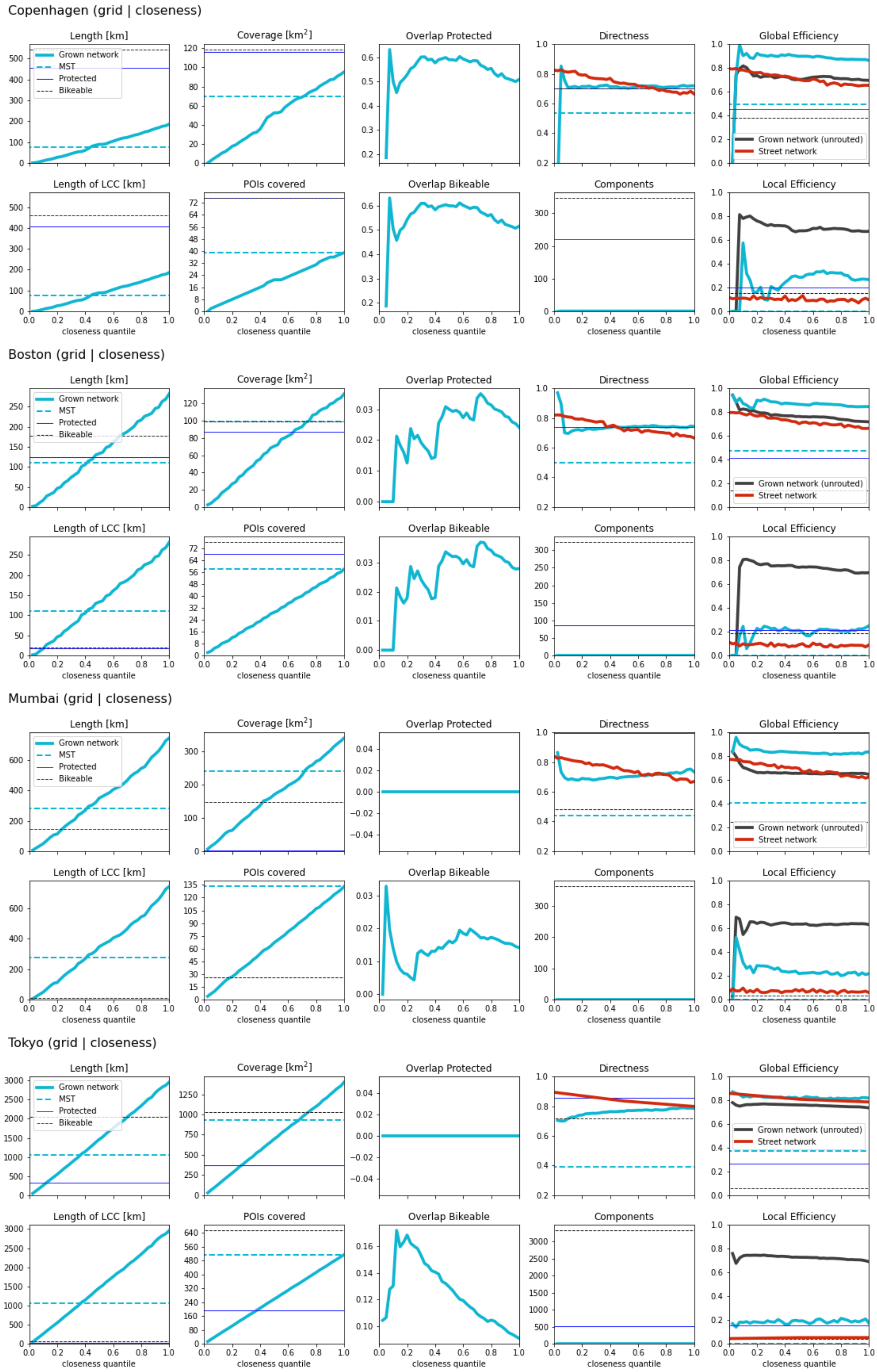

Supplementary Figure 9: **Change of network metrics with closeness growth on grid seeds.** Shown are the selected cities Copenhagen, Boston, Mumbai, Tokyo. See Section Data availability in the main text for how to access these plots for all cities.

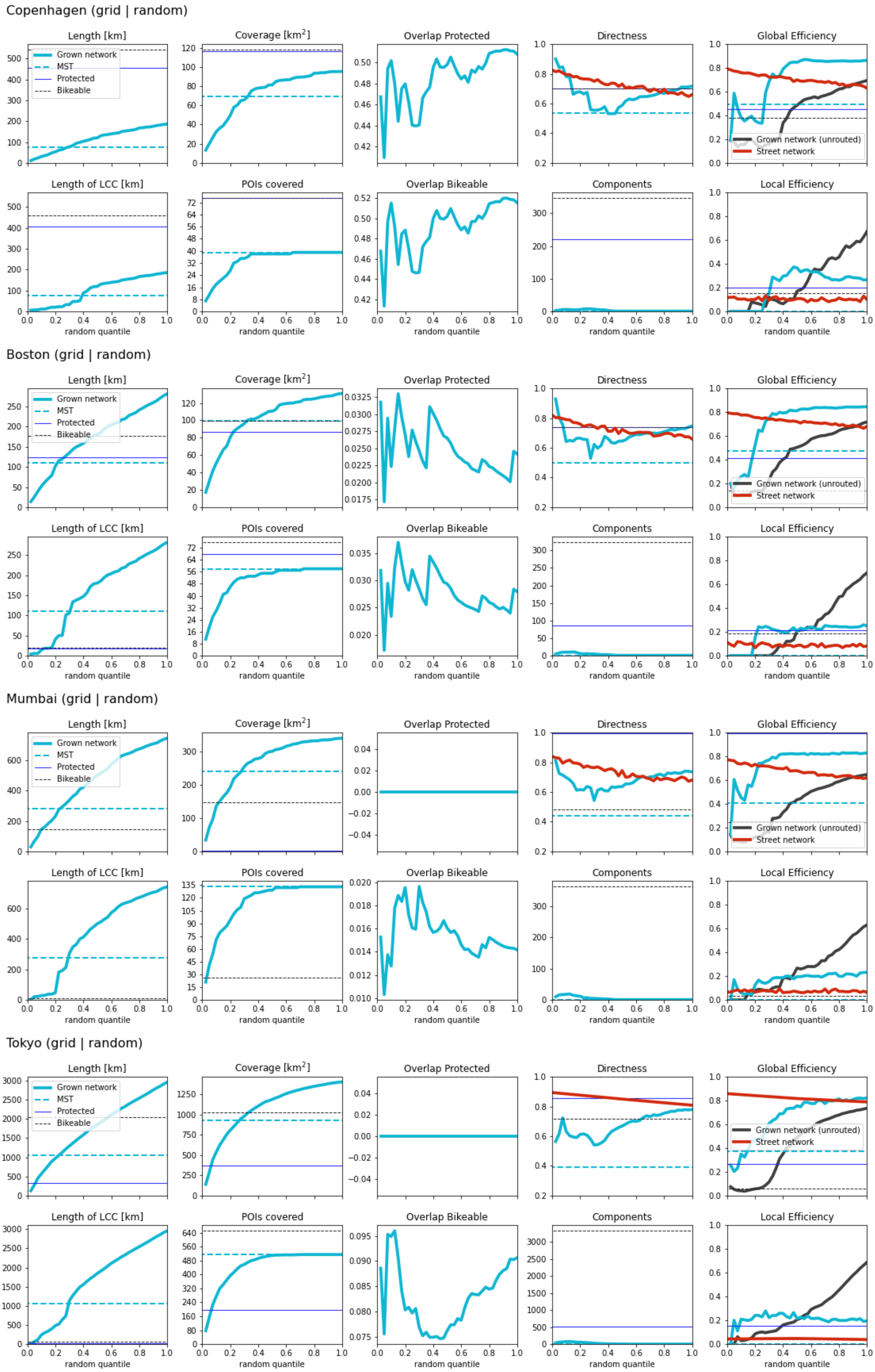

Supplementary Figure 10: **Change of network metrics with random growth on grid seeds.** Shown are the selected cities Copenhagen, Boston, Mumbai, Tokyo. See Section Data availability in the main text for how to access these plots for all cities.
